# Genomic Screening Consortium for Australian Newborns (GenSCAN)

**DOI:** 10.1111/jpc.70131

**Published:** 2025-07-05

**Authors:** Natalie Taylor, Michelle Pirreca, Bruce Bennetts, Gladys Ho, Kirsten Boggs, Karin S. Kassahn, Lucy Anastasi, David Eugeny Godler, Mohammed Alshawsh, Sarah Norris, Joanne Scarfe, Tiffany Boughtwood, Gareth Baynam, Belinda Burns, Enzo Ranieri, Jade Caruana, Sebastian Lunke, Stephanie Best, Zornitza Stark

**Affiliations:** ^1^ School of Population Health, Faculty of Medicine and Health UNSW Sydney New South Wales Australia; ^2^ Sydney Genome Diagnostics, Western Sydney Genetics Program Sydney Children's Hospitals Network Westmead New South Wales Australia; ^3^ Australian Genomics Melbourne Australia; ^4^ Specialty of Genomic Medicine, the Children's Hospital at Westmead Clinical School, Faculty of Medicine and Health University of Sydney Westmead New South Wales Australia; ^5^ Department of Molecular Pathology SA Pathology Adelaide South Australia Australia; ^6^ Adelaide Medical School, Faculty of Health and Medical Sciences The University of Adelaide Adelaide South Australia Australia; ^7^ Murdoch Children's Research Institute Parkville Victoria Australia; ^8^ Department of Paediatrics University of Melbourne Melbourne Victoria Australia; ^9^ Department of Paediatrics Monash University Clayton Victoria Australia; ^10^ The University of Sydney, Faculty of Medicine and Health Sydney School of Public Health, The Leeder Centre for Health Policy, Economics and Data Sydney New South Wales Australia; ^11^ Rare Care Centre, Perth Children's Hospital, Western Australia, Australia; Undiagnosed Diseases Program—WA, Genetic Health Services WA, King Edward Memorial Hospital Perth Western Australia Australia; ^12^ Western Australian Register of Developmental Anomalies, King Edward Memorial Hospital Perth Western Australia Australia; ^13^ Office of Population Health Genomics, Western Australian Department of Health Perth Western Australia Australia; ^14^ NSW Newborn Screening Programme The Children's Hospital at Westmead Westmead New South Wales Australia; ^15^ Faculty of Health and Medical Sciences The University of Adelaide Adelaide South Australia Australia; ^16^ Victorian Clinical Genetics Services, Murdoch Children's Research Institute Melbourne Australia; ^17^ Department of Pathology University of Melbourne Melbourne Australia; ^18^ Melbourne School of Health Sciences University of Melbourne Melbourne Australia

**Keywords:** genetics, genomics, neonatology, newborn screening, rare disease

## Abstract

**Background:**

Using genomic sequencing technology at population scale as a screening test holds the promise of improving outcomes for individuals with rare diseases through early detection and timely access to precision medicine. However, the incorporation of genomics into established newborn screening programmes raises many challenges, ranging from technical feasibility and scalability through to ethical concerns regarding consent and data management. Empirical evidence and implementation experience from large‐scale studies are required to guide future policy.

**Methods:**

We provide a narrative summary of genomic newborn screening studies currently underway in Australia.

**Findings:**

We summarise six research studies currently underway in Australia, which explore the application of genomic technologies in the newborn screening context. These studies have taken varying approaches to generating evidence about the implementation of genomic newborn screening and have formed a national consortium, the Genomic Screening Consortium for Australian Newborns (GenSCAN), with the aim of sharing experiences and enabling collective learning.

**Conclusions:**

Over the next decade, we can expect substantial evidence to be generated nationally and internationally to inform future policy decisions on whether to incorporate genomic sequencing into newborn screening programmes.

**Summary**

- What is already known on this topic?
  ○ Newborn screening (NBS) for rare conditions is a highly effective public health programme.
  ○ It traditionally relies on biochemical markers present in blood
  ○ Incorporating genomic sequencing into NBS has the potential to expand programmes to hundreds of rare conditions.
- What this paper adds?
  ○ Empirical evidence and implementation experience are needed to guide policy
  ○ Six research studies exploring genomic NBS are currently running in Australia.
  ○ The research studies have formed a national consortium to share experience and synthesise evidence.

## 1 Introduction

Newborn bloodspot screening (NBS) is a critical public health intervention that enables early rare disease detection. In Australia, more than 300 000 babies per year have NBS through analysis of a blood sample taken from a baby's heel shortly after birth, with around 300 babies identified annually with a treatable condition [1]. NBS is highly effective for the conditions currently screened, has high uptake, and is delivered at low cost. For example, early NBS diagnosis maximises survival in cystic fibrosis, with 20‐year survival significantly higher in NBS (90%) versus non‐NBS cohorts (70%) [2].

NBS in Australia is delivered by State and Territory Governments and is governed both at State and Territory level and nationally in accordance with the Newborn Bloodspot Screening National Policy Framework. Historically, decisions to add new conditions were made at State and Territory level, leading to some inconsistency in what was screened across Australia. In 2023, all Australian Governments established a formal shared decision‐making process for identifying and deciding on conditions that should be added to, or removed from, NBS. The process involves different stages of condition identification, advice and assessment, with final recommendations provided by all Health Ministers. States and Territories are responsible for implementing any new screening tests once they have been recommended.

Over the past decade, Australian NBS programmes have maintained a relatively consistent approach to the use of technology (typically biochemical approaches) and maintained around 99% nationwide uptake [1]. Currently, 32 target conditions are included in NBS programmes nationally, with a further five conditions recently approved for implementation [3]. Nevertheless, several hundred severe, early‐onset rare conditions are now considered treatable [4] and incorporating genomic sequencing could potentially be used to expand NBS programmes to screen for many of these using a single assay [5]. Genomic sequencing can be used to perform either whole genome sequencing (WGS), which comprehensively evaluates the entire DNA sequence, or targeted genome sequencing, which examines specific regions at a lower cost. Many research studies worldwide are currently piloting the feasibility of integrating genomic sequencing into NBS, using a variety of approaches [5]. The potential challenges of integrating genomics into standard NBS programmes are considerable and have provoked much debate [6]. Key issues include technical feasibility; condition selection and variant interpretation; scalability; cost; public, parental and professional acceptability; consent and data management; privacy and data security concerns; and downstream healthcare system impacts [5]. In addition, the incorporation of genomic sequencing is challenging the established paradigm of NBS programmes (which focus on severe, early‐onset, treatable conditions), with the potential to detect conditions with variable age of onset and severity, including adult‐onset conditions [7], and conditions with no specific treatments available (e.g., neurodevelopmental disorders [8]).

**FIGURE 1:**
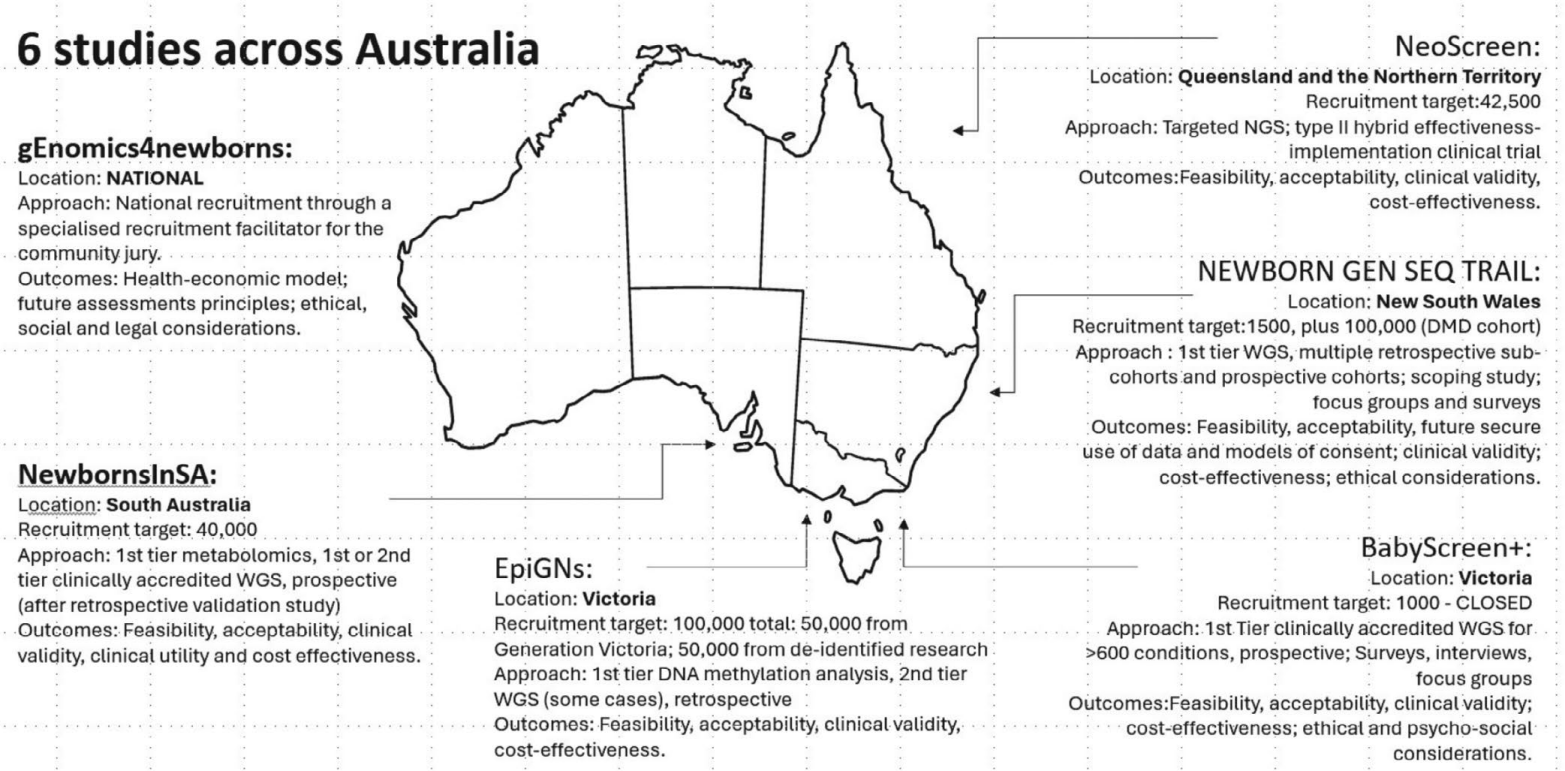
Overview of the six research projects part of the Genomic Screening Consortium for Australian Newborns (GenSCAN). DMD: Duchenne muscular dystrophy; NGS: next generation sequencing; WGS: whole genome sequencing.

The Australian Government has committed to investing $64 million in NBS between 2022 and 2028 to expand the number of conditions screened and improve consistency of delivery across the nation. In addition, in 2022 the Genomics Health Futures Mission (GHFM), the Medical Research Futures Fund (MRFF), and the National Health and Medical Research Council (NHMRC) awarded more than AUD$23 million across six research projects to explore how genomics might be integrated into Australian NBS programmes, Figure 1. The projects are exploring the technical feasibility, clinical utility, and health economic, ethical, and equity aspects of applying first‐ or second‐tier genomic testing in NBS programmes. The research studies have come together to form a national consortium, the Genomic Screening Consortium for Australian Newborns (GenSCAN), to share experiences and enable collective learning. Here, we provide an overview of each of the research studies and of the activities of the GenSCAN consortium. Further information is available in the published study protocols [9, 10, 11, 12] and study websites (see Web resources). All studies are currently active, with the BabyScreen+ study in Victoria having completed recruitment in 2024.

### 1.1 Current Australian Research Projects Exploring the Role of Genomics in NBS

#### 1.1.1 BabyScreen+ (Victoria)

The BabyScreen+ study is a prospective cohort study, assessing the feasibility and acceptability of genomic NBS (gNBS) in 1000 newborns in Victoria, Australia [9]. Prospective parents are approached during the third trimester of pregnancy using a variety of active and passive methods, including as part of consultations with pregnancy care providers (obstetricians, midwives and general practitioners), videos, posters and social media. Enrolment into the study is through an online decision support platform, Genetics Adviser [13], with genetic counselling support available on request. Clinically accredited WGS is performed using DNA extracted from dried blood spot (DBS) cards collected as part of standard NBS, with a target turnaround time to result of 2 weeks. WGS data are analysed for pathogenic and likely pathogenic variants in 605 genes associated with early‐onset, severe but treatable conditions [14]. Low chance results are returned to participants online, with genetic counselling support available on request. High chance results are returned by genetic health professionals, with confirmatory testing and referrals to specialist services organised as required. Carrier status, adult‐onset conditions and variants of uncertain significance are not reported. The design of the study has been informed by focus groups with the Australian public [15, 16] and with health professionals [17]. Participant attitudes towards gNBS and future implementation, including genomic data reuse, will be explored through surveys, interviews and focus groups. In addition, the study will explore a variety of ethical, implementation, psychosocial and health economic factors to inform future service delivery.

#### 1.1.2 Newborn Gen Seq Therapy Ready and Information for Life (TRAIL) Study (New South Wales)

The TRAIL study has eight, predominantly retrospective, cohorts exploring feasibility and acceptability of gNBS as a complement to standard NBS. Working collaboratively with clinicians, TRAIL's retrospective cohorts (comprising 1000 de‐identified DBS cards and samples from 200 patients with a known genetic diagnosis) will be used to test the clinical validity of the 600 plus conditions being considered for inclusion. These retrospective cohorts provide the opportunity to explore technically challenging‐to‐detect conditions, and the inclusion of conditions which may only have clinical trials available. The de‐identified cohort (1000) will be used to demonstrate how gNBS data could be used for other types of genomic screening throughout life, for example carrier screening and polygenic risk scores. To facilitate the re‐use of the data, a single nucleotide polymorphisms (SNP) panel, derived from a second diagnostic specimen, will be used to develop a pathway for confirming data provenance without the need for resequencing [18].

The experience gained through the retrospective cohorts will be used to offer gNBS to a prospective cohort of 100 newborns. Recruitment will be in the third trimester of pregnancy. The study will explore developing models of parental education delivery and consent through a purpose‐built dynamic consent platform, GeneGuardian, developed in collaboration with CSIRO. In addition, TRAIL will also explore the educational needs of the nursing and midwifery workforce.

The TRAIL study is also facilitating a scoping study which is adding screening for Duchenne muscular dystrophy (DMD) to standard NBS in NSW for 12 months. A total of 100 000 newborns will be screened using a two‐tier approach (creatine kinase measurement followed by *DMD* gene sequencing). This scoping study will help understand what evidence is required for a condition to be included in Australian NBS programmes. Through the cumulation of retrospective cohort data, infrastructure development, workforce analysis and pilot studies, the TRAIL study aims to prepare NSW for participation in a future scaled version of gNBS.

#### 1.1.3 NewbornsinSA (South Australia)

The NewbornsInSA research study is exploring a multi‐omics approach to expand NBS, combining metabolomics and WGS [11]. As part of this study, a novel metabolomic screen is being developed that may assist in distinguishing the profiles of newborns with a range of genetic conditions from healthy newborns. Up to 40 000 prospectively recruited newborns can receive metabolomic screening. Of this group, the families of 1000 newborns will be invited to have gNBS, with reporting limited to around 600 conditions that are early‐onset and treatable. The study is being advertised at major birthing hospitals throughout South Australia, pathology collection centres and via social media. Families self‐enrol and consent to genomic testing during pregnancy or shortly after birth; videos and lay‐language study materials support families during the e‐consent process. Newborns with prenatal or postnatal complications may be referred to the study by their healthcare provider and can opt‐in to have WGS. All families will receive their gNBS results. Newborns with a high chance finding will be referred to clinical services for confirmatory diagnostic testing and clinical management. The feasibility and clinical effectiveness of this screening model will be evaluated using the prospective data obtained through the NewbornsInSA research study, including family experiences collected through surveys pre‐ and post‐result. The study will also investigate general public and stakeholder acceptability.

#### 1.1.4 Epi‐Genomic Newborn Screening (EpiGNs) Program (Victoria)

The EpiGNs program is investigating the use of DNA methylation patterns in the detection of a range of disorders caused by changes in epigenetic modification such as Fragile X, Prader‐Willi (PWS), Angelman (AS), Dup15q, Turner, XXY, XXXY, and XXYY syndromes. Clinical trials are currently underway exploring novel therapies for many of these conditions, providing a rationale for early, population‐based ascertainment. The project has developed a novel workflow which is being validated using DBS cards from 50 000 infants recruited as part of Generation Victoria (GenV), a whole‐of‐state birth cohort, and 50 000 infants consented for de‐identified research in Victoria, Australia [10]. EpiGNs uses a two‐tiered approach whereby a DNA methylation screen is performed on an initial DBS card sample, with positive cases analysed using genomic and epigenetic testing on another punch from the DBS card to confirm aetiology. A pilot study performed to date on 16 579 infants [19] from the general population found that: (i) the positive predictive value for cases positive from 2 DBS punches by 1st and 2nd‐tier testing for the conditions screened is ~100%; (ii) the prevalence of PWS and AS is higher than figures reported from clinical ascertainment. The key aims of the study are: (i) to scale the workflow and determine the prevalence of these conditions in 100 000 infants from the general population; (ii) to identify clinically actionable methylation screening thresholds for the first‐tier screen for the conditions screened using phenotypic measures collected up to 3 years of age in ~50 000 infants recruited by GenV. The outcomes of the study will provide an evidence‐based assessment regarding the feasibility of incorporating screening based on DNA methylation patterns, particularly as the treatment for this group of conditions evolves.

#### 1.1.5 NeoScreen (Queensland)

The primary aim of the NeoScreen study is to develop a systematic understanding of how the health system, healthcare professionals, and families may respond to the implementation of a complex clinical intervention like gNBS. In close collaboration with Queensland Health, NeoScreen aims to understand how to appropriately implement and scale genomic technology into NBS. Informed by implementation, psychosocial, and clinical research methods, the study has the following objectives: (1) comprehensive assessment of existing NBS systems and processes, in particular consent for NBS and NBS research activities; (2) mapping and monitoring of gNBS systems integration, and identification of behavioural and organisational factors affecting implementation; (3) identification of clinical, technical, ethical, psychosocial, and legal implications of including specific conditions in gNBS (e.g., for each condition, considerations will include whether there are specialists available for/capable of ongoing management of children with diagnosed conditions); (4) co‐design of an implementation framework for the integration of gNBS; (5) identification of the clinical and cost‐effectiveness of adding specific conditions to NBS via genomic sequencing; (6) design of state‐based adaptations for integration of genomic technology and management of new conditions in NBS. Consent optimisation and implementation are currently underway in Queensland following two national workshops, and operational working groups are driving forward key implementation recommendations prior to piloting implementation of gNBS. These include changes to the DBS card, parent information, staff training, and incorporating research activities.

#### 1.1.6 gEnomics4newborns: Integrating Ethics and Equity With Effectiveness and Economics for Genomics in Newborn Screening (Nationwide)

It is well‐recognised that integrating genomics into existing NBS programmes brings with it a range of important economic, ethical, legal and social considerations which require input from a wide range of stakeholders to inform future public health policy in this area. While the other Australian studies are investigating these aspects to some degree, they are the primary focus of the gEnomics4newborns research project [12]. The project is employing a mix of research methods including: qualitative (interviews with parents of children with a rare condition, health professionals, scientists and policy makers), quantitative (equity‐informed economic modelling, and a stakeholder choice experiment), and deliberative (a national Citizens' Jury with members of the general public, and Yarning Circles with Australian Indigenous people) to explore the legal, ethical, equity, and economic implications of the use of genomics in NBS. Equity is a particularly important aspect in the context of the genetically diverse population of Australia, and the availability of universal, government‐funded access to healthcare for all Australians. The findings from this project will be used to develop policy‐relevant tools that will be designed to support future NBS decision‐making by government.

### 1.2 Genomic Screening Consortium for Australian Newborns (GenSCAN)

**FIGURE 2:**
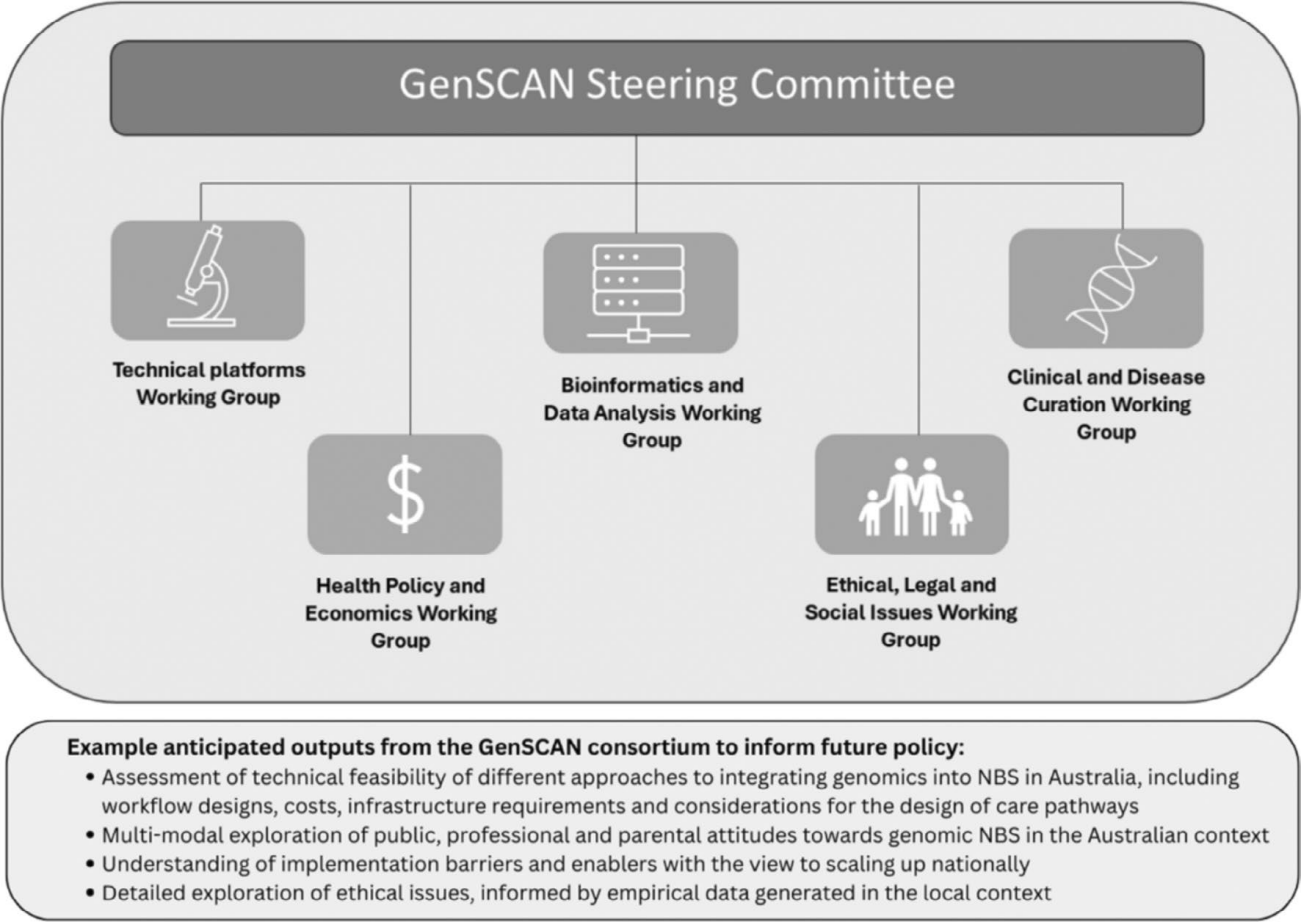
Structure of the GenSCAN Consortium: Steering Committee, Working Groups and anticipated outputs.

The GenSCAN consortium brings together the six research studies currently running in Australia with the aim of collating and synthesising the evidence generated to inform possible future uses of genomics in Australian NBS programmes. There are five working groups overseen by a Steering Committee, Figure 2. The role of the working groups is to facilitate shared learning and knowledge exchange between the projects, reducing duplication of effort and working towards future harmonisation. For example, in the planning stages of the studies, the Clinical Working Group considered which conditions may be suitable for inclusion and what criteria should be used in decision‐making [20]. The list of conditions developed by the BabyScreen+ study [14] has been adopted with modifications by the NewbornsInSA study and is being appraised by the TRAIL study in New South Wales, thus avoiding duplicative effort. As the studies have progressed to enrolling and testing newborns, the Clinical Working Group has set up a national virtual multi‐disciplinary team meeting to discuss challenging data interpretation issues. This includes discussions about how to assess the pathogenicity of genetic variants in the screening context and developing consensus reporting thresholds and practices. Similarly, the Bioinformatics Working Group has explored technical challenges in data analysis, potential solutions and practical experiences of developing screening workflows including automation, whereas the ELSI Working Group has facilitated discussion around appropriate consent models, in terms of timing (in pregnancy or after birth) and modality (in‐person or using digital platforms). As the studies near completion over the next 2 years, the GenSCAN consortium will synthesise the evidence generated across the projects and provide this information to policy makers.

### 1.3 Outlook

While the delivery of NBS programs remains the responsibility of the Australian States and Territories, the need for a nationally consistent approach is well recognised. If, when and how to include more genomic sequencing in the NBS programs will continue to be debated nationally and internationally as our understanding of the clinical, economic and policy aspects of gNBS evolves. The current round of research studies will generate important evidence regarding technical feasibility, screening yields, and acceptability. While this local evidence can be collated with the results from other studies running concurrently in healthcare systems globally, additional information from larger‐scale, longer‐term local projects will be needed to inform decisions regarding implementation at population scale. In addition to generating evidence, such large‐scale studies also serve to prepare the healthcare system by establishing infrastructure, building capacity and developing downstream healthcare pathways. Genomic technologies are also being considered for other types of population screening, notably in healthy adults, for example to inform personal or reproductive risk. It is therefore crucial that nationally consistent approaches are developed for genomic population screening, regardless of life stage. As these programs scale, wide stakeholder engagement and broad engagement with the Australian public will be critical to inform the evolution of policy regulations and legislation in line with evidence, and public and professional expectations. Given NBS is a critical and effective public health measure, if gNBS is implemented it must be done in a manner that maintains the current high levels of participation without creating or widening disparities in health outcomes for screened newborns.

## Ethics Statement

All the studies described hold relevant institutional ethics as follows: gEnomics4newborns (Sydney Childrenʼs Hospital Network HREC 2023/ETH02371; University of Wollongong Social Sciences HREC 2024/241; The University of Sydney HREC 2024/HE000854); TRAIL study (Sydney Childrenʼs Hospital Network HREC 2023/ETH01674, 2023/ETH02532, 2023/ETH02489, 2024/ETH00618, 2024/00314, 2024/ETH00673, 2024/ETH01978); EpiGNs program (Royal Childrenʼs Hospital Melbourne HREC/92777/RCHM‐2023 v2); NeoScreen (The University of New South Wales HREC 2024/HE000561, 2024/iRECS5688); NewbornsinSA (Womenʼs and Childrenʼs Health Network HREC 2022/HRE00258, 2023/HRE00236); BabyScreen+ (Royal Childrenʼs Hospital HREC/91500/RCHM‐2023, HREC/90929/RCHM‐2022 and HREC/91392/RCHM‐2022).

## Conflicts of Interest

The authors declare no conflicts of interest.
